# A Guide to Molecular Characterization of Genotype II African Swine Fever Virus: Essential and Alternative Genome Markers

**DOI:** 10.3390/microorganisms11030642

**Published:** 2023-03-02

**Authors:** Ali Mazloum, Antoinette van Schalkwyk, Roman Chernyshev, Alexey Igolkin, Livio Heath, Alexander Sprygin

**Affiliations:** 1 Federal Center for Animal Health, 600901 Vladimir, Russia; 2 Agricultural Research Council-Onderstepoort Veterinary Institute, 100 Old Soutpan Road, Onderstepoort 0110, South Africa; 3 Department of Biotechnology, University of the Western Cape, Robert Sobukwe Road, Bellville 7535, South Africa

**Keywords:** African swine fever, marker, gene, differentition, phylogeny

## Abstract

African swine fever is a contagious viral disease that has been spreading through Europe and Asia since its initial report from Georgia in 2007. Due to the large genome size of the causative agent, the African swine fever virus (ASFV), the molecular epidemiology, and virus evolution are analyzed by employing different markers. Most of these markers originate from single nucleotide polymorphisms or disparities in the copy number of tandem repeat sequences observed during the comparisons of full genome sequences produced from ASFVs isolated during different outbreaks. Therefore, consistent complete genome sequencing and comparative analysis of the sequence data are important to add innovative genomic markers that contribute to the delineation of ASFV phylogeny and molecular epidemiology during active circulation in the field. In this study, the molecular markers currently employed to assess the genotype II ASFVs circulating in Europe and Asia have been outlined. The application of each of these markers to differentiate between ASFVs from related outbreaks is described to implement a guideline to their suitability for analyzing new outbreaks. These markers do not signify the complete repertoire of genomic differences between ASFVs, but will be beneficial when analyzing the first outbreaks in a new region or a large number of samples. Furthermore, new markers must be determined via complete genome sequence analyses for enabling in-depth insights into the molecular epidemiology of ASFV.

## 1. Introduction

African swine fever virus (ASFV) is the only member of the genus *Asfivirus*, of the family *Asfarviridae* [1]. The virus is the causative agent of African swine fever (ASF), a disease identified by hemorrhagic fever with a lethality rate of up to 100% in domestic pigs and wild boars. It constantly infects natural hosts, warthogs, and bush pigs, as well as soft ticks of the genus Ornithodoros (*O. moubata*, *O. erraticus*), which serve as a biological vector of virus transmission [2].

The virus encapsulates a double-stranded linear DNA genome of ~189 kilobase pairs (kbp) that encodes more than 180 putative open reading frames (ORFs). The genome length and number of ORFs can vary based odatan the virus strain or genotype. Yet, notwithstanding the genotype, the genome is segmented into three main regions. The central conservative region is ~125 kbp and has less than 1.5% size differences from all the known ASFV sequences. It is flanked by two highly variable terminal regions [3]. Currently, ASFVs are classified into 24 genotypes based on the variable C-terminal region of the *B646L* gene, encoding for the structural protein p72 [4,5].

At present, the disease is deemed endemic to several countries, yet following its initial outbreak in East Africa in 1909 and for the ensuing decades, it was restricted to the African continent [6]. In 1957, outbreaks of ASFV genotype I were first observed in different European countries, whilst in the 1970s the same genotype I was found across the Atlantic in the Caribbean islands [7]. By the 1990s, the virus was eliminated in all of these countries except for Sardinia, where ASFV genotype I is constantly circulating [8]. A new introduction of ASFV, known as genotype II, was reported in Georgia in 2007 [9]. It had a dramatic and prolific spread to Armenia, Azerbaijan, Russia, Ukraine, Moldova, and the European Union, starting with eastern Europe and then to the central parts reaching Belgium and Italy. During recent years (2015–2022), ASF cases and outbreaks were reported in several European countries (the Czech Republic, Germany, Hungary, Italy, Latvia, Poland, the Republic of North Macedonia, Romania, Bulgaria, Lithuania, Serbia, Slovakia, and Estonia). In all EU countries except for Romania, wild boar was the major affected species [10]. The spread of ASFV included southeast Asia, where in 2018 it was observed in China, Mongolia, and Vietnam [10]. The disease was again detected in the Caribbean archipelago with outbreaks reported in the Dominican Republic and Haiti in 2021; the causative ASFVs belonged to genotype II [11]. In 2021, genotype I was reported in China, which was genetically comparable to the ASFV influencing the 1960′s outbreaks in Europe [12].

This pandemic influenced by genotype II ASFVs had a massive impact on the swine industry and the global economy. At least in Europe, wild boars are the core disease transmission factor, but the transmission between wild boars (WB) and domestic pigs (DP) remains unknown; however, the human factor may play a major role in virus transmission. As a method of choice, scientists have been using several genome markers to identify and determine samples collected from WB and DP. These markers are sequences of either partial genes or intergenic regions. Unfortunately, the markers chosen to determine innovative ASFV samples vary between countries and laboratories, with the exclusion of the *B646L* gene used for genotyping. This inconsistent usage of markers complicates the decision of which marker(s) to use when studying new isolates, causing a misrepresented picture of the global ASF epidemiology. Furthermore, the well-established markers described to determine ASFVs’ belonging to genotype I, such as I196L, KP86R, I78R/I215L, and E183L, were inadequate when applied to genotype II ASFVs, which indicates an objective need for universal loci [13].

This study seeks to offer a guide to the selection and application of markers for the subsequent characterization of ASFV genotype II samples linked to the Georgia/2007 isolate. The published primers and protocols for each of the genome markers, as well as a classification of the numerous variants collected by each of the different markers, are outlined. The markers are classified into two groups: essential markers for subtyping ASFVs and additional markers.

## 2. Essential Genetic Markers Used for Subtyping Genotype II ASFVs

Given the fast and prolific spread of genotype II ASFVs, from the first discovery in 2007 in Georgia to both Europe and Asia, this clonal virus population has accrued various mutations discovered by determining and evaluating the complete genome sequence of different isolates [14]. Notwithstanding the valuable information acquired from identifying the complete genome sequences, this process is time-consuming, labor intensive, and costly, and is subsequently replaced by the fast and affordable amplification and sequencing of multiple selected markers. This technique enables the molecular characterization of large sample numbers from multiple outbreaks, contributing to the comprehension of the disease epidemiology. Yet, it is still recommended to conduct NGS sequencing of the complete viral genomes over time or from outbreaks in new location, to identify innovative markers [14].

Identification of the connection between different field samples depends solely on the genetic characterization of specific genomic regions. Restriction fragment length polymorphism analysis (RFLP) was the first method employed to distinguish the samples based on their polymorphisms, but this method was time-consuming and difficult to apply [15,16,17]. RFLP protocols have been substituted by PCR amplification and Sanger sequencing of selected regions containing informative nucleotide polymorphisms. All the markers outlined in this review are based on the latter two technologies.

**1. *B646L* : Genotyping marker.**

**Figure 1:**
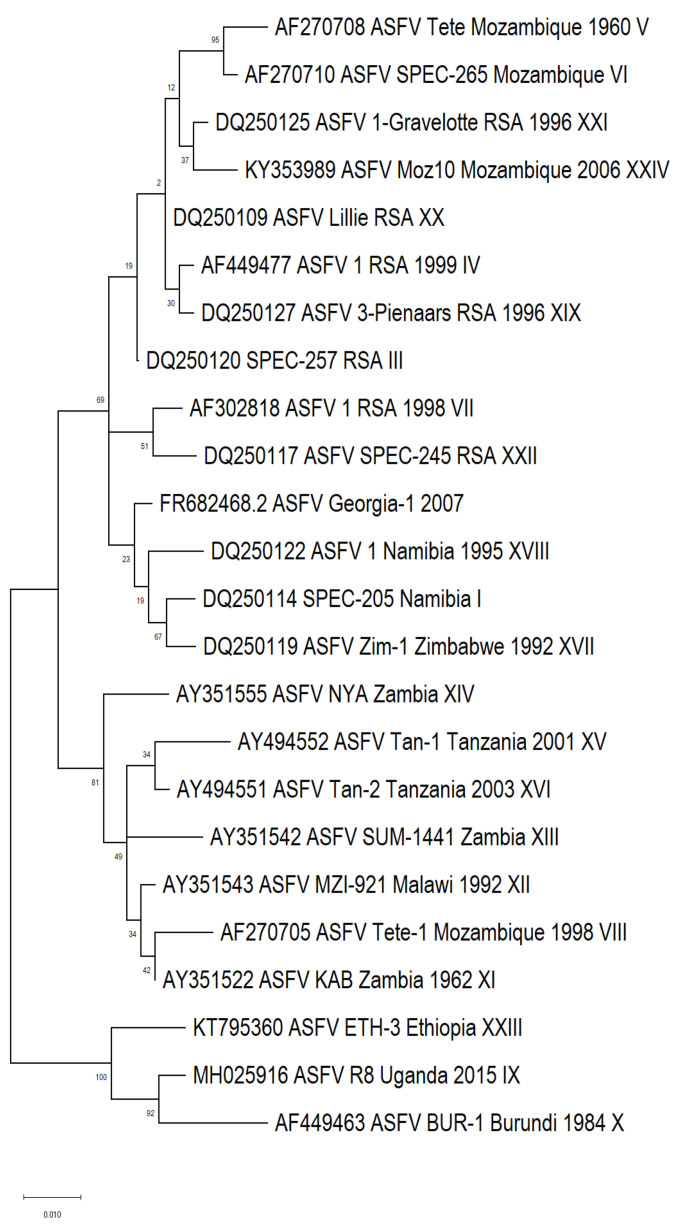
Phylogenetic tree based on partial sequence of C-terminal region of *B646L* gene, representing all 24 genotypes of ASFV, using reference isolates from GenBank.

In 2002, Bastos et al. described the first protocol of using PCR amplification and Sanger sequencing of the variable C-terminal region of the *B646L* gene to classify ASFVs into genotypes [4]. Currently, 24 genotypes of ASFV have been detected, with the last description from Ethiopia [18]. The phylogenetic connection of the 24 genotypes is highlighted in Figure 1.

To optimize the C-terminal region of gene *B646L*, encoding the major structural virus protein vp72, the oligonucleotide primers P72-F (5′ GGC ACA AGT TCG GAC ATG T 3′) and P72-R (5′ GTA CTG TAA CGC AGC ACA G 3′) are proposed [4]. The resulting amplicon is 478 bp and both primers P72-F and P72-R can be applied in the subsequent Sanger sequencing reactions. It is crucial to compare the consensus sequence, acquired from assembling both forward and reverse sequences, with other sequences denoting all 24 genotypes. A phylogenetic tree produced with the assembled sequence can be employed to determine the genotype of the new samples. Information regarding isolates representing all 24 genotypes, as well as the subsequent accession numbers acquired from GenBank, are presented in Supplementary Table S1.

Evaluation of the concatenated sequences of both *B646L* and *B602L* gene regions estimated the substitution rate as 3.31 × 10^−4^/site/year. Despite the high substitution rate, these genes retained substantial nucleotide conservation to be applied during the genotype determination of ASFVs [19]. This genome marker is important for the differentiation of ASFVs into genotypes, but its resolution does not go beyond the genotype, especially when applied to genotype II populations currently circulating in Eurasia. This is because of the recent introduction of the common ancestor of these genotype II ASFVs. This limitation requires the use of alternative genetic regions of the viral genome, comprising considerable variation to further differentiate between new ASFV samples within genotype II.

**2. Central variable region (CVR) of *B602L* gene: Sub-genotyping marker.**

After the initial genotyping of novel ASFVs, the CVR sequence of each sample is established for sub-genotyping. The CVR of the ASFV genome is confined within the *B602L* gene and is about 372 bp in length. The predicted translated sequences of the CVR comprise several tandem tetramers, including CADT, NADT, NVDT, and CASM. Analysis of the percentage and composition of tandem tetramer repeats confined within the CVR of an ASFV sample can be used to determine and/or group multiple ASFV samples [20].

Due to the difference in the number of tandem tetramer repeats, the CVR amplicons may vary in length between 228 and 686 bp [21,22]. Since the number of tandem tetramers within one genotype may vary between samples, the CVR cannot be utilized for genotyping, but rather as an additional tool for the characterization or sub-genotyping of the samples.

**Figure 2:**
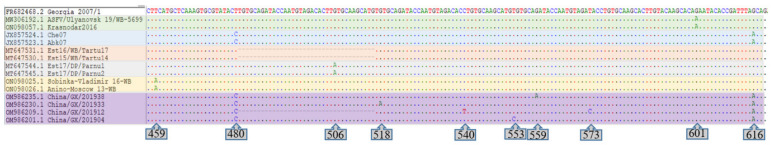
Nucleotide sequence alignment of the partial CVR gene. The six different groups identified in Europe and the RF are expressed in different colors, whilst the four variants unique to China are expressed in violet color.

Several *B602L* subgenotypes have been outlined within the genotype II ASFVs circulating in Eurasia. Three variants of the virus were discovered in Estonia, with the first group sharing 100% sequence similarity with Georgia 2007/1. The second variant had a 35 bp deletion at position 481, influencing an amino acid deletion of CASM CADT NVDT, whilst the third variant comprised a non-synonymous (A/G) single nucleotide polymorphism (SNP) at position 506, which resulted in a cysteine (C) to tyrosine (Y) exchange at the predicted amino acid position (position 193) of the complete *B602L* protein (Figure 2) [23].

In the Russian Federation (RF), another three variants were detected, in addition to the large cluster that shares 100% sequence identity with Georgia 2007/1. The first variant had an A/G mutation at position 601, influencing a lysine (K) exchange of a glutamic acid (E) at the predicted amino acid position 201 of the *B602L* protein (Figure 2). The second group of variants was characterized by a single synonymous (A/T) SNP at position 459 and was signified by samples obtained between 2013 and 2016 in the RF (Figure 2). The last group of variants was prevalent between samples from the RF, Armenia, Azerbaijan, and Ukraine and was characterized by two unique SNPs in contrast to the reference sequence Georgia 2007/1 (FR682468.2). These SNPs were synonymous (C/T) at position 480 and A/G at position 616. The latter SNP resulted in a threonine (T) exchange of alanine (A) at position 206 of the complete *B602L* predicted protein [24]. An alignment of the partial CVR (*B602L*) nucleotide sequence of different isolates indicating each of the six groups is depicted in Figure 2.

Samples from South Korea and Vietnam shared 100% sequence identity with Georgia 2007/1 and were thus clustered within subgroup I [25,26]. However, a recent analysis of 66 ASFVs circulating in the Guangxi Province of China between 2019 and 2020 demonstrated high heterogeneity of the CVR sequences, with four variants detected within this region that are explicit to China (Figure 2, violet color) [27].

**Figure 3:**
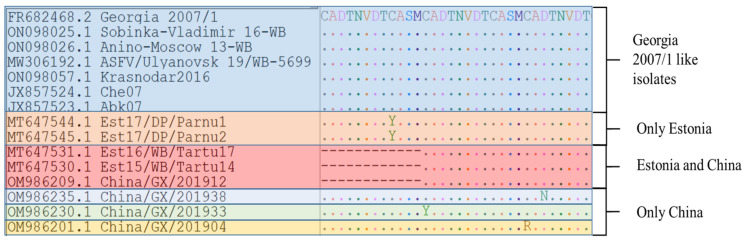
Amino acid sequence alignment of the tetrameric tandem repeat sequences (TRSs) of the central variable region (CVR) of the *B602L* gene.

Two additional non-synonymous SNPs were outlined in samples from Poland and Lithuania in 2017 [28]. Since these sequences are not available in GenBank and were only outlined in these two unrelated outbreaks, they were not included in either of the analyses of Figure 2 and Figure 3.

Gray indicates the position of SNPs based on the bottom strand of the genome (distance from the start codon since this gene, *B602L*, is reversed). Positions were determined according to the original gene *B602L* of ASFV isolate Georgia 2007/1. Geographic distribution of all ten identified variants based on the partial sequencing of CVR of isolates from Europe and Asia are represented in Supplementary Figure S1A.

Furthermore, the ASFV genotype II samples could be classified based on the predicted AA sequence of the CVR, which comprise the three tetramers “CADT”, “NVDT”, and “CASM.” The number of repeats varied between the various samples; for example, Georgia 2007/1, the reference isolate, had eight copies of these tetramers (CADT NVDT CASM CADT NVDT CASM CADT NVDT). In the EU and Asia, based on the AA tetramer, six groups have been determined so far, with one group being unique to Estonia, three groups unique to China, and one group common between China and Estonia (Figure 3).

To expand the CVR region and evaluate either nucleotide or amino acid sequence, it is suggested to use primer pair CVR-Fwd (5′ AAG CTC ATT AGG CAC ATT TAA TGT TTT TTG C 3′) and CVR-Rvs (5′ CTG CAG GAA TGG ATG CCT TC 3′) with annealing temperature 60 °C. The same primers were employed during Sanger sequencing of the amplicon [20].

**3. Intergenic region *I73R*/*I329L*: Sub-genotyping marker.**

The intergenic region (IGR) located between ORFs *I73R* and *I329L* has recently been identified as a standard method for subtyping genotype II ASFVs from Europe and Asia [29]. The Georgia 2007/1 (FR682468.2) sequence comprises two copies of the tandem repeat sequences (TRS) in the IGR *I73R*/*I329L*, leading to the reference group designation of IGR-I.

In 2014, Gallardo et al. characterized an additional insertion of the 10 bp TRS (GAATATATAG) in the ASFVs from Ukraine, Belarus, Lithuania, and Poland, resulting in these sequences being called IGR-II [30]. The same insertion was determined in the complete genome sequence of strain Odintsovo 02/14, an isolate from the RF, in 2014 [31]. Subsequently, samples with three TRS (IGR-II sub-genotype) have been co-circulating with sub-genotype IGR-I in the EU, Russia, and Asia. However, since 2014, the majority of isolates analyzed in the EU have belonged to IGR-II, making it the dominant variant. This was further defined by Gallardo C. et al., 2023 when 367 samples from ASF wild boar cases and pigs outbreaks submitted between 2012 and 2022 from the EU were evaluated for this region and it was revealed that 95.91% of the isolates belonged to IGR-II [28].

**Figure 4:**
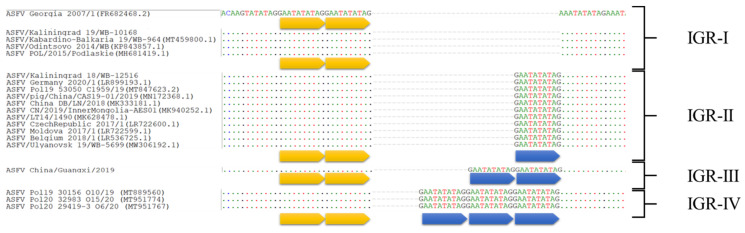
Nucleotide alignment of ASFV samples based on the sequences of the intergenic region (IGR) between *I73R* and *I329L*. Each arrow denotes one TRS; yellow arrows indicate TRSs identical in quantity to the reference isolate Georgia 2007/1; blue arrows represent additional TRS.

The initial identification of IGR-III, comprising four copies of the 10-nucleotide TRS, as outlined in the sequence, China/Guangxi/2019, was isolated from a domestic pig in the Guangxi Province of China in 2019 [32]. In January 2021, samples from Vietnam were found to cluster in both IGR subgenotypes (IGR-I and IGR-II), but in February 2022 four ASFV isolates from Vietnam were discovered to belong to IGR-III (Figure 4) [33,34,35].

A fourth cluster (IGR-IV) was revealed, containing five 10 nt TRS repeats, in the eastern Warmińsko-Mazurskie voivodeship of Poland [36]. This locus contributes significantly to the study of samples from Germany, Latvia, Estonia, Lithuania, and the Kaliningrad region of Russia [37]. Geographic distribution of all four identified variants based on the partial sequencing of IGR of isolates from Europe and Asia are represented in Supplementary Figure S1B.

It is proposed to use the primer set, ECO1A (5′ CCA TTT ATC CCC CGC TTT GG 3′ targeting positions 172,270–172,290) and ECO1B (5′ TCG TCA TCC TGA GAC AGC AG 3′ binding site 172,616–172,626), to optimize an approximately 356 bp fragment located between the *I73R* and *I329L* genes [30]. Another pair of primers expanding a 349 bp in the same IGR was proposed in 2020 by Mazur-Panasiuk et al., IGR-F (5′ CTC AGA ACT TTT TGA GAA GAT TG 3′ binding site 172,236–172,258) and IGR-R (5′ CAG CAA ACA GTC CTA TTG TT 3′ binding site 172,585–172,566) [36]. The same primers were employed in subsequent Sanger sequencing of the amplicons and the copy number of 10 bp (GAATATATAG) TRS was identified.

The position of primers depicted here is identified based on the whole-genome sequence of reference isolate Georgia 2007/1 (accession number # FR682468.2).

**4. Intergenic region *MGF-505-9R*/*10R*: Sub-genotyping marker.**

An innovative 17 bp TRS (GATAGTAGTTCAGTTAA) insertion in the IGR between ORFs *MGF-505-9R* and *MGF-505-10R* was explained in seven samples from the Tver, Vladimir, and Smolensk regions of the RF in 2016, and nine isolates from Poland, unlike the Georgia/2007 sequence [29,38]. Subsequently, it was implied that the copy number and type of TRS could vary between isolates, resulting in eight separate groups [28]; unfortunately, these sequences signifying those eight groups were not all published in GenBank. It is proposed to use primers MGF-Fwd (5′ AGA AAC CGC AGA TGA ATG TA 3′) and MGF-Rvs (5′ TAC AGC CCT AGT TGT TGA AG 3′) at an annealing temperature of 55 °C during the amplification of this intergenic region [39]. The same primers were employed in subsequent Sanger sequencing of the amplicons and the number of 17 bp TRS (GATAGTAGTTCAGTTAA) insertions were assessed.

**5. *K145R*: Sub-genotyping marker.**

**Figure 5:**
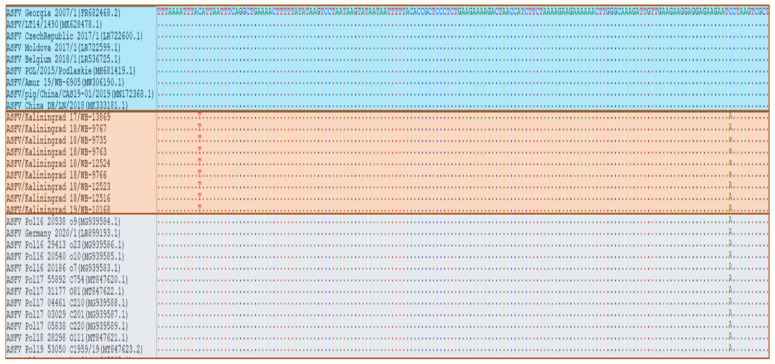
Nucleotide alignment of ASFV samples based on the partial sequence of the *K145R* gene and depicting only three groups. Blue color—group *K145R-I*, which is similar to Georgia 2007/1; gray color—group *K145R-II* isolates with one SNP (C-to-A transversion at position 434); orange color—group *K145R-III* isolates with two simultaneous SNPs (C > T at position 291 and C > A at position 434).

The first comprehensive genome sequences of ASFV isolates from Poland suggested a C-to-A transversion at position 434 of the *K145R* gene, unlike the Georgia 2007/1 sequence [40]. This SNP was further observed in sequence Germany/2020 (LR099193). Sequence analysis of the partial *K145R* gene of 154 ASFVs from Poland facilitated the differentiation of two subgroups: *K145R-I* and *K145R-II*. These sequences were clustered as (*n* = 124) belonging to cluster *K145R-II* (with the SNP), whilst the remaining (*n* = 30) were comparable to Georgia 2007/1 in cluster K145-I (Figure 5) [36].

Sequence analysis using the complete *K145R* gene of isolates from the Kaliningrad region of the RF observed two simultaneous SNPs, one unique to the sequences from the Kaliningrad region in comparison with Georgia/2007 and the second comparable to the SNP discovered previously in Poland and Germany [36,40]. The first SNP (C > T at position 291) was unique to sequences from Kaliningrad and was called the cluster *K145R-III*, whilst the second SNP (C > A at position 434) was similar to cluster *K145R-II* as previously reported in the EU (Figure 5) [36,37]

It was suggested to use primers *K145R*-Fwd (5′ TTT CAG GCT GAA AAC TTT TTA T 3′) and *K145R*-Rvs (5′ AAA GTT TTC AAT GGT TGT TAG C 3′) with annealing temperature 55 °C to optimize the partial *K145R* gene [40]. However, since these primers yield an amplicon that covers only one SNP (at position 434), a new primer pair was developed. The primers New-*K145R*-Fwd (5′ GCA GCT TTA CCG CAG CAT AC 3′) and New-*K145R*-Rvs (5′ AAG AGT AGG TGG GCG CTT TC 3′) amplify a 501 bp region of the *K145R* gene using an annealing temperature of 55 °C.

**6. *O174L* : Sub-genotyping marker.**

**Figure 6:**
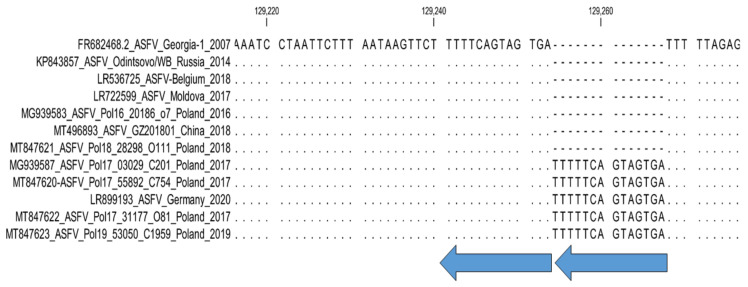
Nucleotide alignment of ASFV samples based on the partial sequence of the *O174L* gene, demonstrating the two groups. Group *O174L-I* is identical to Georgia 2007/1, whilst group *O174L-II* contains two 14nt TRS.

A new 14-nucleotide TRS (TCACTACTGAAAAA) was observed in the *O174L* gene, encoding for the DNA polymerase-X protein, of 12 isolates originating from Poland were found in 2019 [41]. A sequencing study of an additional 154 samples from WB and DP reported between 2017 and 2020 in Poland determined the additional 14 nt TRS insertion in 50 of the samples (Figure 6) [36]. A similar insertion was determined in the complete genome sequence of strain Germany/2020, isolated from a wild boar in Germany in 2020, and recently in 19 ASFV samples from Romania [28,42].

The integration of markers described in genes *O174L* and *K145R* is important to evaluate the spread and molecular epidemiology of genotype II ASFVs in Germany, Poland, and other eastern European countries [28,36,42].

It is proposed to use primers *O174L*-Fwd (5′ TGG CTC AGA CGA TAT TTC AAC TC 3′) and *O174L*-Rvs (5′ GCC TCC ACC ACT TGA ACC AT 3′) with annealing temperature 55 °C during the amplification of the *O174L* gene. The same primers were employed during the subsequent Sanger sequencing of the amplicons and the number of 14-nucleotide TRS (TCACTACTGAAAAA) was identified [36].

## 3. Additional Sub-Genotyping Markers

NGS sequencing of samples from new outbreaks yields more genomic data about ASFV evolution. Genomic markers are established based on the analysis of complete genome sequences that are subsequently used to subgroup strains of genotype II ASFVs. Since these markers were outlined during complete genome analyses, not all of them have specific primers suggested for the amplification and Sanger sequencing of the following regions. The description of the following markers should enable researchers to design primers and create future assays.

**1. Intergenic region A179L/A137R: Sub-genotyping marker.**

**Figure 7:**
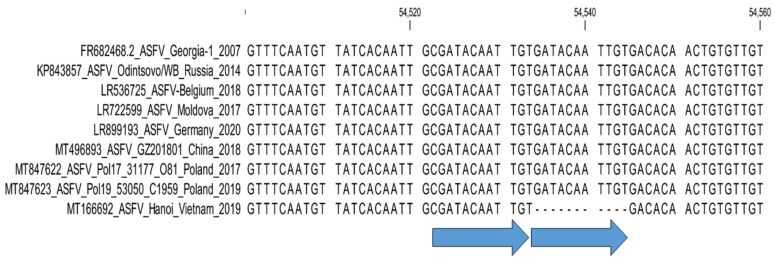
Nucleotide alignment of ASFV samples using the intragenic regions between ORFs *A179L* and *A137R* genes. Sample ASF_Hanoi_Vietnmam_2019 [MT166692] has only one copy of the 11 bp TRS sequence, compared with the two copies in Georgia 2007/1.

An 11-nucleotide deletion (GATACAATTGT) within the IGR between the ORFs *A179L* and *A137R* genes was determined in two ASFV strains (ASFV/VN/Pig/Hanoi/07 and ASFV_Hanoi_2019) from Hanoi city in Vietnam [39]. Intriguingly, the «ASFV/VN/Pig/Hanoi/02» strain that is intimately linked to «ASFV/VN/Pig/Hanoi/07» had this 11-nucleotide repeat, as well as all other strains of genotype II ASFVs. Ongoing research focusing on evaluating this marker is needed to determine if these variants are confined to circulation in Vietnam and China only (Figure 7).

It is proposed to use primers *A179L*/*A137R*: forward (5′ CCA TAG CGG CAC CCT ATA TT 3′) and reverse (5′ CCT CCT GGT CGA GTT TGG TA 3′) with annealing temperature 50 °C during the amplification of this intergenic region [39]. The same primers were utilized in subsequent Sanger sequencing of the amplicons and the presence or absence of the 11 bp (GATACAATTGT) region was identified.

**2. *MGF-505-5R* and *MGF-110-7L*: Sub-genotyping markers.**

The G-to-A transversions in both genes *MGF-505-5R* and *MGF-110-7L*, unlike the reference strain Georgia 2007/1, were initially described in the analysis of the complete genome sequence of an ASFV isolate from Poland [40]. This SNP at position 988 in *MGF-505-5R* was non-synonymous and facilitated the exchange of valine (V) with isoleucine (I) at position 303 of the predicted protein. This substitution was revealed in an additional 57 of the 72 samples acquired from Poland between 2017 and 2020 [36].

**Figure 8:**
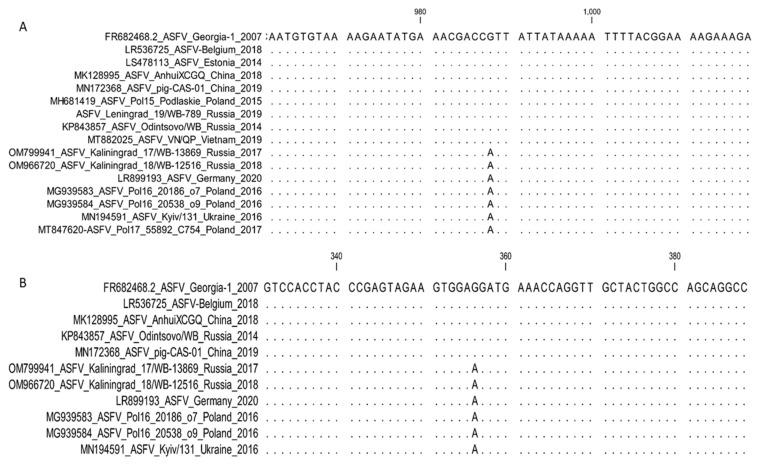
Nucleotide alignment of ASFV isolates based on the analysis of the partial sequence of (**A**) *MGF-505-5R* and (**B**) *MGF-110-7L*, showing the SNP in these regions among genotype II isolates.

The same transversion at position 60 in the *MGF-110-7L* gene was synonymous with the predicted protein [40]. The strains from Germany and Ukraine, Germany/2020 (LR099193) and Kyiv/131 2016 (MN194591), comprised similar SNPs in both genes as were characterized in the sequences from Poland. Furthermore, both the SNPs identical to Poland were revealed when analyzing the complete genome sequences of nine ASFV isolates from the Kaliningrad region of the RF, sampled between 2017 and 2019 (Figure 8).

Thus, both the markers found in *MGF-505-5R* and *MGF-110-7L* can be employed to distinguish between ASFV samples circulating in eastern Europe, Asia, and the RF, exempting the Kaliningrad enclave from ASFVs found in western Europe.

For the analysis of MGF 505_5R, it is suggested to use the following primers: MGF-505-5R-F (5′ TAC GCT TCT TTT CAA TCA TCA T 3′) and MGF-505-5R-R (5′ AAA TTA ACA GTT GTT TGC CTT C 3′) with annealing temperature 50 °C [36].

Currently, no specific primers are suggested for the amplification and sequencing of *MGF-110-7L*, since the observations were based on the analysis of complete genome sequences (Figure 8).

**3. *I267L*: Sub-genotyping marker.**

**Figure 9:**
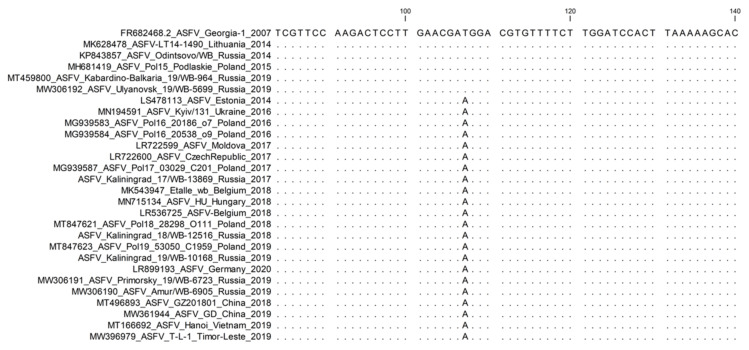
Nucleotide sequence alignment of ASFV samples using the partial ORF *I267L* gene. Samples from Poland, China, and the east of Russia contain an “A,” whilst the reference sequence Georgia 2007/1 and the western region of the RF contain a “T”.

A non-synonymous T-to-A transversion SNP was discovered within the *I267L* gene (position 583), resulting in an Ile-to-Phe exchange at position 195 of the predicted protein. This SNP was initially observed in the complete genome sequence of ASFV, isolated between 2016 and 2019 from Poland [40]. The identical substitution was verified for isolates from Ukraine, Moldova, European countries (Estonia, the Czech Republic, Belgium, Hungary, and Germany), all isolates sequenced from China, and the Far Eastern Federal District of Russia (Figure 9) [14,43,44,45,46].

It is proposed to use primers *I267L*-Fwd (5′ TTG GAC AAA TTG CGT TGC GA 3′) and *I267L*-Rvs (5′ AAA TGC GAC CGT CCA GAA CT 3′) with annealing temperature of 55 °C to amplify and sequence the partial *I267L* gene.

**4. *MGF-360-10L* and *MGF-505-9R*: Sub-genotyping markers.**

**Figure 10:**
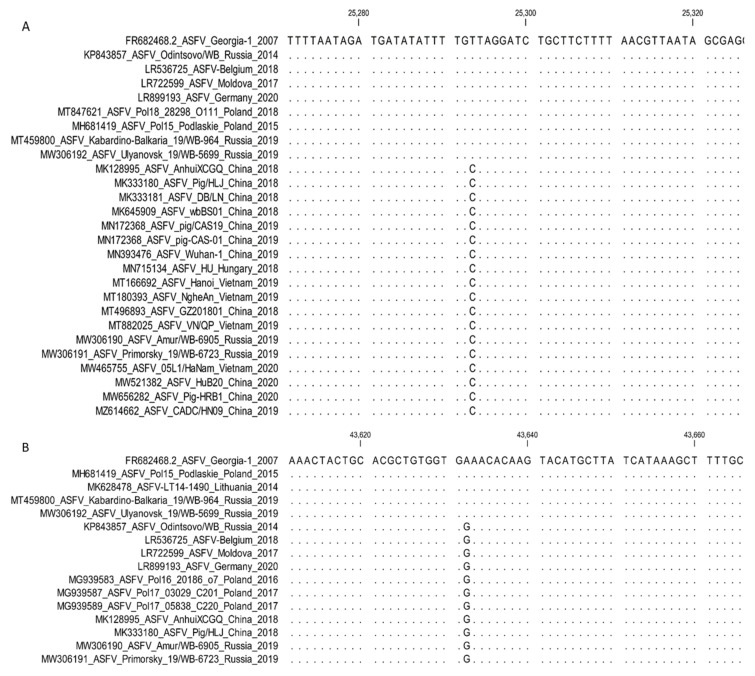
Nucleotide sequence alignment of ASFV samples using the partial ORFs *MGF-360-10L* (**A**) and *MGF-505-9R* (**B**) genes. A. The T-to-C substitution in the *MGF-360-10L* gene at position 677 from the starting codon of the gene. B. An A-to-G substitution in the *MGF-505-9R* gene at position 967 from the starting codon of the gene.

The SNPs observed in these genes were based on complete genome sequence analysis of three isolates from China (ASFV/Anhui/XCGQ/China/2018 [MK128995], ASFV-SY18 [MH766894.2], Pig/HLJ/2018 [MK333180], and DB/LN/2018 [MK333181]) against Georgia/2007. These SNPs were a T-to-C substitution in the *MGF-360-10L* gene at position (677) and an A-to-G substitution in the *MGF-505-9R* gene at position (967) (Figure 10) [43]. Both substitutions were non-synonymous and led to alterations in the predicted amino acid sequence (Asn 329 Ser in *MGF-360-10L* and Lys 323 Glu in *MGF-505-9R*). These SNPs were detected in five ASFV isolates from the Far Eastern Federal District of Russia (ASFV/Primorsky/19/WB-6723 [MW306191], ASFV/Amur/19/WB-6905 [MW306190], ASFV/Zabaykali/2020/WB-5314 [MZ325862], and ASFV/Zabaykali/2020/DP-4905 OP510033]) [14,44,45,46].

**5. Intergenic region *C315R*/*C147L*: Sub-genotyping marker.**

**Figure 11:**
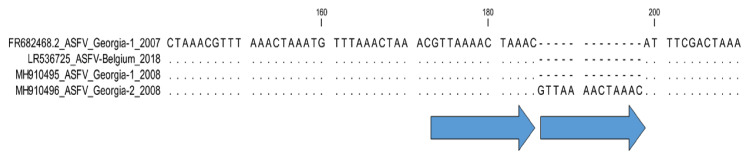
Nucleotide alignment of ASFV samples using the intragenic regions between ORFs *C315R* and *C147L*. Sample Georgia 2008/2 [MH910496] has two copies of the 13 bp TRS sequence, compared with the single copy in Georgia 2007/1.

Comparative analysis of the complete genome sequences of ASFV isolates from Georgia in 2008 (Georgia 2008/2 [MH910496]) compared with 2007 (Georgia 2007/1 [FR682468]) implied an additional fourth TRS (GTTAAAACTAAAC) between the genes *C315R* and *C147L* (Figure 11) [47]. In contrast, the sequences of Georgia 2007/1 and Georgia 2008/1 [MH910495] had only three TRS in the intergenic region *C315R*/*C147*L. Since this TRS was identified once and based on full genome sequencing data, further investigations of this possible marker in other genotype II isolates are needed to determine its resolution and application to assess the molecular evolution of ASFV.

## 4. Conclusions

The spread of ASFV across Europe and into Asia has been rapid, vast, and in the face of no effective vaccine, it will probably keep expanding its range into new countries globally [48]. While spreading across Eurasia, the clonal populations of the virus accrue mutations that can be common in a region and used for evaluating ASFV radiation events [24,30,41]. In this regard NGS promotes the generation of complete genome sequences and can be employed to track the virus from one outbreak to another, identifying innovative variants and assigning sequences to a specific variant group [14,26,37]. Unfortunately, identifying the complete genome sequence of an isolate is expensive, time-consuming, and requires specialized equipment and skills. A substitute to this process is to assess strains from novel outbreaks based on the amplification and Sanger sequencing of chosen genomic markers, provided the markers contain adequate polymorphisms to elucidate the molecular epidemiology of an outbreak. Marker-based methods are time- and cost-efficient and allow for the processing of large proportions of samples from either a single outbreak or multiple outbreaks. Currently, there is not a single genomic marker capable of discerning between the different genotype II ASFVs circulating in Europe and Asia, but collectively multiple genomic loci are showing improvement to assess the molecular epidemiology of these outbreaks [28].

Novel markers are constantly being described through in-depth comparisons of the complete genome sequences of samples acquired from the outbreak [14,37,40]. It is therefore crucial that regular sequencing and analysis of the complete ASFV genomes be conducted to assess the efficacy of the current markers. Unfortunately, the description of each new marker indicates more PCRs and Sanger sequences that are required to segment and investigate the molecular epidemiology of an outbreak in the aspect of previous samples. It is therefore beneficial to examine the possible application of other technologies than PCR and Sanger sequencing for multiplexing the chosen markers.

Fluorescent probe-based multiplex real-time PCR technology has been determined and applied to the field of molecular diagnostics. Recently, a multiplex real-time quantitative PCR assay was outlined for the detection of ASFV and simultaneous discrimination between virulent and multi-gene deleted candidate vaccine strains [48]. Real-time PCR can be applied as an alternative technique to SNP-based differentiation between isolates. Unfortunately, the proportion of variables that could be assessed in a single reaction was restricted by the capabilities of the thermocycler.

Alternative fluorescent real-time PCR techniques that are not probe-based include the discrimination abilities of high-resolution melting assays. This technique could be beneficial to distinguish between the multiple markers containing multiple copies of TRS, such as the CVR and *O174L* ORFs; the IGRs between *I73R*/*I329L*, *MGF-505-9R*/*10R*, and *C315R*/*C147L*; as well as the markers containing an SNP. Amplicons of markers that vary in melting temperature could be distinguished using denaturing gradient gel electrophoresis. We presume that these technologies will make good use of the identified SNPs and support the ASFV differentiation efforts in a cost- and time-efficient manner.

Additional research is currently underway to determine a cost-effective method for simultaneous analysis of these markers to circumvent the process of conducting multiple individual PCRs and sequencing reactions. Unfortunately, the latter remains the only approach currently available to assess the markers outlined in this study.

## Data Availability

All data reported in this study are available within the manuscript and in GenBank.

## Supplementary Materials

The following supporting information can be downloaded at: https://www.mdpi.com/article/10.3390/microorganisms11030642/s1.
