# Supplementary material for: A Guide to Molecular Characterization of Genotype II African Swine Fever Virus: Essential and Alternative Genome Markers
Source: Microorganisms. 2023 Mar 2;11(3):642. doi: 10.3390/microorganisms11030642 (PMC10056344; doi:10.3390/microorganisms11030642)
Supplement: Supplementary file 1 [file microorganisms-11-00642-s001.zip › microorganisms-2212794-supplementary.pdf]

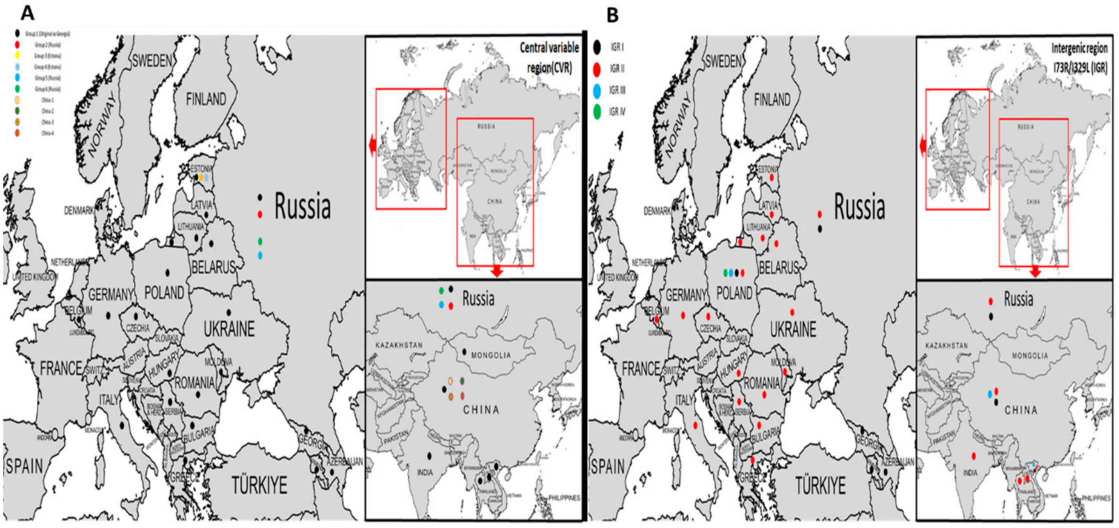

Supplementary Figure S1. Geographic distribution of ASFV variants in Europe and Asia based on the partial sequencing of CVR (B602L gene)—(**A**), and the Intergenic region I73R/I329L (IGR)—(**B**). Number and size of dots do not reflect the number of outbreaks.

Supplementary Table S1. List of 24 ASFV isolates used in the phylogenetic analysis to confirm the respective genotypes.

Accession number	Isolate name	Country	Genotype
DQ250114	SPEC-205	Namibia	I
FR682468.2	Georgia 2007/1	Georgia	II
DQ250120	SPEC/257	South Africa	III
AF449477	RSA 1/1999	Thabazimbi/South Africa	IV
AF270708	Tete	Mozambique	V
AF270710	SPEC-265	Mozambique	VI
AF302818	RSA 1/1998	South Africa	VII
AF270705	Tete-1	Mozambique	VIII
MH025916	R8	Uganda	IX
AF449463	BUR-1	Burundi	X
AY351522	KAB	Zambia	XI

AY351543	MZI-921	Malawi	XII
AY351542	SUM-1441	Zambia	XIII
AY351555	NYA	Zambia	XIV
AY494552	Tan-1	Tanzania	XV
AY494551	Tan-2	Tanzania	XVI
DQ250119	Zim-1	Zimbabwe	XVII
DQ250122	NAM 1/1995	Windhoek, Namibia	XVIII
DQ250127	3-Pienaars	South Africa	XIX
DQ250109	Lillie	South Africa	XX
DQ250125	1-Gravelotte	South Africa	XXI
DQ250117	SPEC-245	South Africa	XXII
KT795360	ETH-3	Ethiopia	XXIII
KY353989	Moz10	Mozambique	XXIV

Supplementary Table S2. Primer sequences previously described for the amplification and sequencing of ASFV genotype II markers.

Marker	Primer	Sequence (5′–3′)	Position	Fragment length (bp)	Referenc e
B646L (genotyping)	P72-F	GGCACAAGTTCGGACATGT	104,581–104,599	478	[4]
	P72-R	GTACTGTAACGCAGCACAG	105,040–105,058		
CVR of B602L gene	CVR-Fwd	AAGCTCATTAGGCACATTTAATGTTTTTT GC	102,591–102,621	459	[30]
	CVR-Rvs	CTGCAGGAATGGATGCCTTC	103,030–103,049		
Intergenic region I73R/I329L (IGR)	ECO1A	CCATTTATCCCCCGCTTTGG	172,272–172,291	356	[30]
	ECO1B	TCGTCATCCTGAGACAGCAG	172,608–172,627		
	IGR-F	CTC AGA ACT TTT TGA GAA GAT TG	172,236–172,258	349	[36]

	IGR-R	CAG CAA ACA GTC CTA TTG TT	172,585–172,566		
Intergenic region MGF-505 9R/10R	MGF-Fwd	AGAAACCGCAGATGAATGTA	45,079–45,098	552	[38]
	MGF-Rvs	TACAGCCCTAGTTGTTGAAG	45,611–45,630		
Intergenic region A179L/A137R	Forward	CCATAGCGGCACCCTATATT	55,295–55,314	270	[39]
	Reverse	CCTCCTGGTCGAGTTTGGTA	55,545–55,564		
K145R	K145R-Fwd	TTTCAGGCTGAAAACTTTTTAT	66,015–66,036	283	This study
	K145R-Rvs	AAAGTTTTCAATGGTTGTTAGC	66,276–66,297		
O174L	O174L-Fwd	TGGCTCAGACGATATTTCAACTC	129,152–129,174	673	[36]
	O174L-Rvs	GCCTCCACCACTTGAACCAT	129,805–129,824		
MGF-505-5R	MGF-505-5R-F	TACGCTTCTTTTCAATCATCAT	38,942–38,963	641	[36]
	MGF-505-5R-R	AAATTAACAGTTGTTTGCCTTC	39,561–39,582		
I267L	I267L-Fwd	TTGGACAAATTGCGTTGCGA	170,704–170,723	337	This study
	I267L-Rvs	AAATGCGACCGTCCAGAACT	171,021–171,040		
